# Distal femoral varus deformity becomes a cause of undercorrection in open wedge high tibial osteotomy

**DOI:** 10.1002/jeo2.70621

**Published:** 2026-01-13

**Authors:** Akira Maeyama, Tetsuro Ishimatsu, Taiki Matsunaga, Kotaro Miyazaki, Yoshiaki Hideshima, Takuaki Yamamoto

**Affiliations:** ^1^ Department of Orthopaedic Surgery, Faculty of Medicine Fukuoka University Fukuoka Japan

**Keywords:** femur, knee joint, osteotomy, regression analysis, tibia

## Abstract

**Purpose:**

To evaluate whether a high lateral distal femoral angle (LDFA) contributes to undercorrection of lower‐limb alignment following medial open wedge high tibial osteotomy (MOWHTO), and to determine the LDFA threshold above which tibial correction alone may be insufficient.

**Methods:**

This retrospective study analysed 68 patients who underwent MOWHTO at our hospital between 2020 and 2022. Based on their postoperative mechanical axis (MA), patients were categorised into two groups: the acceptable correction group (A‐group; MA 57%–67%) and the undercorrection group (U‐group; MA ≤ 56%). Radiographic parameters before and after surgery were compared between the two groups. Simple and multiple regression analyses were used to identify factors affecting postoperative MA. A receiver operating characteristic (ROC) curve was utilised to identify the LDFA cut‐off value associated with undercorrection.

**Results:**

A total of 54 patients met the inclusion criteria (A‐group, *n* = 35; U‐group, *n* = 19). The U‐group demonstrated a significantly higher preoperative mechanical LDFA (89.5° vs. 87.6°, *p* = 0.001) and a lower medial proximal tibial angle (mMPTA) (84° vs. 86°, *p* = 0.014) than that in the A‐group. Both mLDFA (*p* = 0.006) and mMPTA (*p* = 0.010) were independent predictors of postoperative MA in multiple regression analysis. The ROC analysis identified an LDFA cut‐off of 89.2° for predicting undercorrection (area under the curve = 0.78; sensitivity, 36.8%; specificity, 88.6%). Patients with an LDFA ≥ 89.2° had significantly higher body mass index, joint line convergence angle, varus stress angle, percentage condylar tibial translation and preoperative knee joint line obliquity.

**Conclusions:**

A high LDFA (≥89.2°) is associated with an increased risk of undercorrection after MOWHTO, despite accurate tibial correction. These findings suggest that in cases with significant distal femoral varus, isolated tibial osteotomy may be insufficient. Double‐level osteotomy, including femoral correction, should be considered in such cases to achieve optimal alignment.

**Level of Evidence:**

Level Ⅳ, retrospective comparative study.

**Abbreviations**
AJLO: ankle joint line obliquity
AUC: area under the curve
A‐group: acceptable correction group
BMI: body mass index
CTF: condylar tibial translation
HAA: hindfoot alignment angle
ICC: intraclass correlation coefficient
JLCA: joint line convergence angle
KJLO: knee joint line obliquity
KL: Kellgren–Lawrence classification
LDFA: lateral distal femoral angle
MA: mechanical axis
mLDFA: mechanical lateral distal femoral angle
mMPTA: mechanical medial proximal tibial angle
MOWHTO: medial open‐wedge high tibial osteotomy
OA: osteoarthritis
ROC: receiver operating characteristic
U‐group: undercorrection group

## INTRODUCTION

Medial open‐wedge high tibial osteotomy (MOWHTO) is a widely accepted surgical technique for managing medial compartment osteoarthritis (OA) of the knee, especially in younger and more active patients [3, 13, 20, 21, 22]. This procedure adjusts lower limb alignment from varus to mild valgus, thereby redistributing the load across the knee joint, reducing medial compartment pain and postponing the need for knee arthroplasty [6, 7, 8]. Achieving the desired correction relies heavily on accurate preoperative planning and precise adjustment of the mechanical axis (MA), with a postoperative MA target of approximately 62% across the tibial plateau considered optimal for clinical outcomes [7, 9]. While favourable outcomes have been reported when accurate correction is achieved, both undercorrection and overcorrection remain clinically relevant issues [4, 11, 14, 26].

Postoperative alignment errors are multifactorial and arise from the complex three‐dimensional osteoarticular configuration of the lower limb [11, 14, 19]. Unlike correction of a single long bone, MOWHTO must account for adjacent joints and surrounding soft tissues [17, 22]. Variability in posture, ligament laxity, intraoperative conditions and imaging techniques can all affect alignment accuracy [13, 21, 22]. Additionally, changes between weight‐bearing preoperative imaging and non‐weight‐bearing intraoperative positioning may contribute to alignment discrepancies [25, 27].

In addition to these factors, increasing attention has been paid to the role of femoral morphology, particularly distal femoral varus deformity [10, 19, 26]. A high mechanical lateral distal femoral angle (mLDFA) has been proposed as a potential cause of undercorrection following MOWHTO, even when the intended tibial correction is achieved [10, 12]. However, the mechanism by which a high LDFA leads to residual varus alignment remains unclear. It is plausible that in patients with significant distal femoral varus, tibial valgus correction alone may be insufficient to achieve optimal postoperative limb alignment.

Therefore, this study aims to investigate the contribution of distal femoral morphology to residual varus alignment after MOWHTO, and to define an LDFA threshold where isolated tibial correction may be insufficient.

## METHODS

### Study design and ethical approval

This retrospective, nonrandomized, sequential case review was conducted at our institution after receiving approval from the Institutional Review Board (approval no. H23‐10‐003). All participants provided informed consent prior to their inclusion in the study.

### Patient selection

Between 2020 and 2022, a total of 68 patients who underwent MOWHTO for isolated medial compartment knee osteoarthritis were initially considered for this study. All procedures were performed at a single institution by experienced orthopaedic surgeons following a standardised surgical protocol.

### Inclusion and exclusion criteria

#### Inclusion criteria

– Failure of at least 6 months of conservative treatment.
– Isolated medial compartment knee osteoarthritis (Kellgren–Lawrence grade II–IV).
– Varus deformity confirmed radiographically.
– Range of motion: minimum knee extension ≤5° flexion up to 120°.
– High preoperative activity level (e.g., UCLA Activity Score ≥6).

#### Exclusion criteria

– History of prior lower limb trauma or surgery (*n* = 1).
– Postoperative overcorrection (mechanical axis deviation beyond planned correction) (*n* = 4).
– Incomplete or missing preoperative/postoperative radiographic data (*n* = 3).
– Abnormal subtalar joint compensation, defined as hindfoot alignment angle (HAA) ≥ 15.9° (*n* = 6).

**Figure 1:**
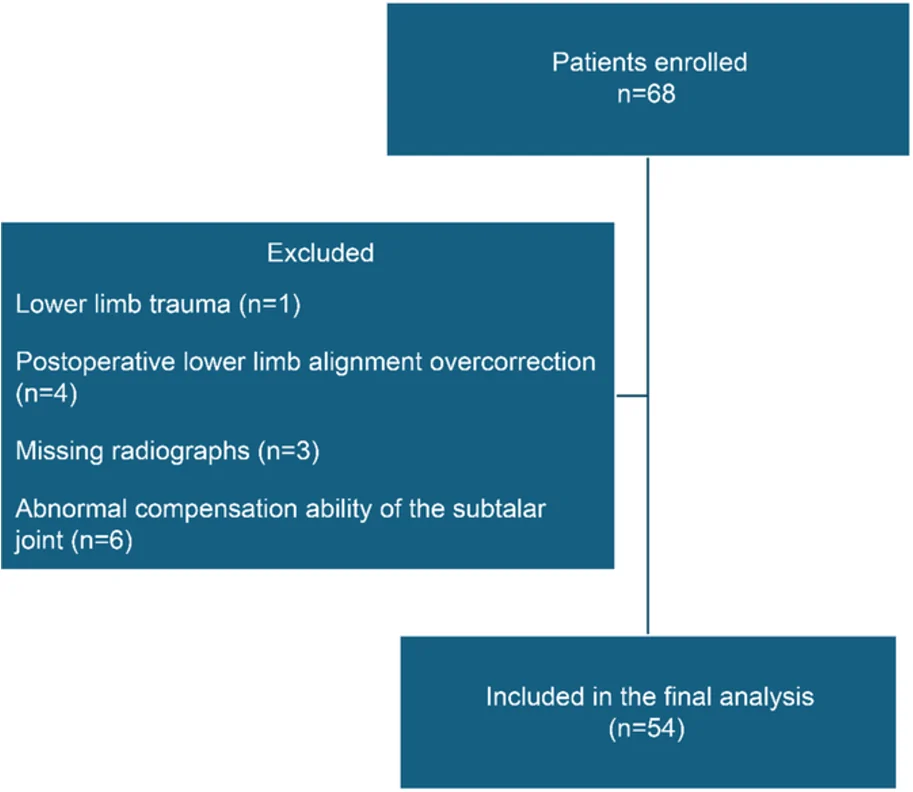
Patient enrolment flowchart.

After applying these criteria, 54 patients were included in the final analysis (Figure 1). All patients completed the required radiographic and clinical follow‐up through the end of the study period.

#### Surgical technique and postoperative care

In preoperative planning, the target weight‐bearing line was set at the Fujisawa point (MA: 62%) on the standing ling‐leg anteroposterior radiograph [8]. The required correction angle was initially calculated using Miniaci′s method [16]. Final adjustments to the correction angle were made based on Ogawa′s method [21], in which JLCA under varus stress was considered. If the JLCA was ≥6°, the correction angle was reduced by 0.3° for each degree of JLCA, accounting for soft tissue correction.

A cut was made toward the proximal tibiofibular joint and was stopped approximately 5 mm from the lateral cortical edge. Next, a cut was made in the frontal plane. The cut was gradually opened using a spreader until the planned correction angle was achieved. The medial gap was then filled with biphasic tricalcium phosphate (b‐TCP) blocks to support the osteotomy site. The medial side was stabilised with a locking plate (TriS Plate®; Olympus Terumo Biomaterials). Postoperative rehabilitation included isometric quadriceps exercises and active foot joint motion starting on postoperative Day 3. Patients began straight leg raises and unrestricted continuous passive motion. Partial weight‐bearing (50%) was allowed starting on postoperative Day 2, with full weight‐bearing permitted from postoperative Day 4.

### Radiographic evaluation

Standardised, full‐length anteroposterior weight‐bearing radiographs of the lower limbs were obtained preoperatively and at 6 months postoperatively. All images were acquired with the knees fully extended and the patellae facing anteriorly to ensure consistent positioning.

**Figure 2:**
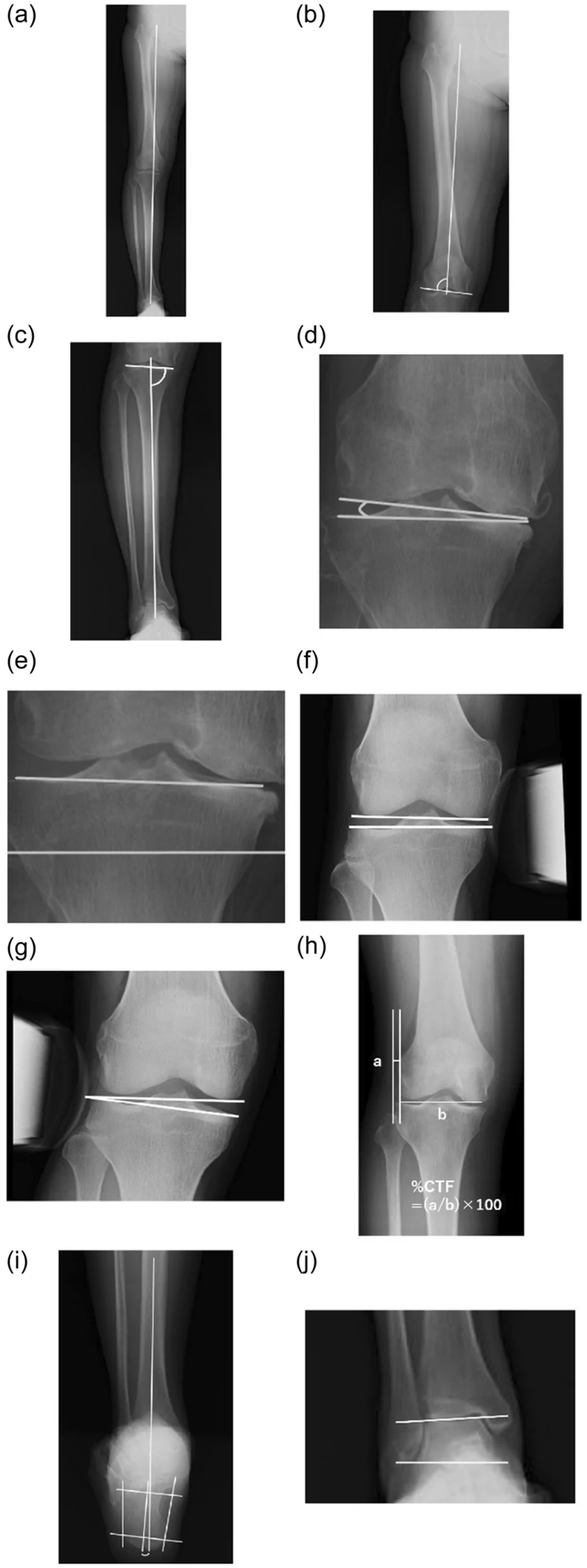
Radiographic evaluation parameters: mechanical axis (MA, a), mechanical lateral distal femoral angle (mLDFA, b) medial proximal tibial angle (mMPTA, c), joint line convergence angle (JLCA, d), knee joint line obliquity (KJLO, e) and ankle joint line obliquity (AJLO). Panels (f) and (g) depict varus and valgus stress radiographs under 15 kg load in full extension, respectively, used to assess laxity angles and JLCA under dynamic conditions. Panel (h) illustrates coronal tibiofemoral (CTF) subluxation, expressed as the percentage lateral displacement (%CTF) between femoral and tibial condyles relative to the tibial plateau width. (i, j) Hindfoot alignment angle and AJLO. Negative angular values indicate valgus orientation in KJLO and AJLO assessments.

The following radiographic parameters were assessed:

- MA was measured as the angle between the mechanical axes of the femur and tibia (Figure 2a).
- mLDFA and medial proximal tibial angle (mMPTA) were recorded to evaluate coronal alignment of the femur and tibia, respectively (Figure 2b,c).
- The joint line convergence angle (JLCA), representing the angle between the femoral and tibial joint lines, was assessed under both neutral and stress‐loaded conditions (Figure 2d).
- Knee joint line obliquity (KJLO) was defined as the angular deviation of the tibial plateau from the horizontal axis. Negative values indicated a valgus tilt (Figure 2e).
- Ankle joint line obliquity (AJLO) was similarly assessed using the talar surface as a reference, with negative values denoting valgus deviation (Figure 2e).

To assess joint laxity, stress radiographs were taken in full extension under 15 kg of applied varus (Figure 2f) and valgus (Figure 2g) force, allowing evaluation of varus/valgus laxity angles and JLCA under loading.

Coronal tibiofemoral (CTF) subluxation was quantified by measuring the lateral offset between the femoral and tibial condyles. This distance, normalised to the tibial plateau width, was expressed as a percentage (%CTF). Osteophytes were excluded from measurement landmarks (Figure 2h).

For hindfoot alignment, standardised hindfoot‐view radiographs were used to measure the hindfoot angle (Figure 2i) and AJLO, from which the Hindfoot Alignment Angle (HAA) was calculated as the sum of their absolute values (Figure 2j). Based on prior literature [14, 15], cases with an HAA ≥ 15.9° were excluded due to presumed abnormal subtalar joint compensation.

Patients were categorised into two groups based on postoperative MA alignment: those with acceptable correction (A‐group, MA range 57%–67%, *n* = 35) and those with undercorrection (U‐group, MA ≤ 56%, *n* = 19) [7, 18].

### Statistical analysis

A comparative analysis was conducted between the groups to evaluate both preoperative and postoperative parameters, including age, sex, body mass index (BMI), KL grade, MA measurements, correction angle, mMPTA, mLDFA, JLCA, KJLO, varus/valgus stress angle and percentage condylar tibial translation (%CTF).

The predictors of postoperative MA were investigated using simple and multiple regression analyses. A receiver operating characteristic (ROC) curve analysis was used to determine the optimal preoperative LDFA cut‐off associated with potential undercorrection. Patients were subsequently grouped based on whether their preoperative mLDFA was above or below the derived cut‐off. Statistical comparisons were performed using Student′s *t*‐test for normally distributed variables and the Mann–Whitney *U* test for non‐normally distributed ones, as determined by the Shapiro–Wilk test.

Radiographic measurements were independently performed by two orthopaedic surgeons. Intra‐ and inter‐observer reliability were evaluated using intraclass correlation coefficients (ICC), yielding values ranging from 0.82 to 1.00 and from 0.81 to 1.00, respectively. All statistical analyses were conducted using SPSS software (version 23.0, IBM Corp.).

## RESULTS

Among the 54 patients included in the study, the mean age was 61.5 years, with a slight female predominance (32 females, 22 males). The majority of patients had advanced osteoarthritis, with 94% (51 out of 54) classified as KL grades III or IV. Patients were divided into two groups based on their postoperative MA: A‐group, MA 57%–67% (*n* = 35), and U‐group, MA ≤ 56% (*n* = 19). Comparative analysis of demographic and preoperative radiographic parameters between the two groups showed no significant differences in age, sex distribution, BMI, or KL classification (Table 1). Comparison of preoperative radiographic parameters revealed that the U‐group had a significantly lower preoperative mMPTA (84° vs. 86°, *p* = 0.014) and higher mLDFA (89.5° vs. 87.6°, *p* = 0.001) than that in the A‐group. Other preoperative variables, including MA, correction angle, JLCA, KJLO, varus/valgus stress angles, and %CTF, did not differ significantly between the groups. Postoperatively, the A‐group demonstrated a significantly higher MA (64% vs. 54%, *p* < 0.001), while no significant differences were observed in other postoperative parameters (Table 2).

**Table 1:** Comparison of demographic and radiographic parameters between U‐group and A‐group.

	U‐group	A‐group	*p* value
Total patients (*n* = 54)	*n* = 19	*n* = 35	
Age (years)	60 (55–73)	62 (52–77)	0.294
Sex (male/female)	11/8	11/24	0.345
Body mass index (kg/m^2^)	27.6 (22.7–32.7)	25.6 (20.6–31.1)	0.168
Kellgren–Lawrence grade (Ⅰ/Ⅱ/Ⅲ/Ⅳ)	(0/4/8/7)	(0/11/24/0)	0.105

**Table 2:** Comparison of preoperative and postoperative radiographic parameters between U‐group and A‐group.

	U‐group		A‐group	*p* value
Preoperative				
MA (%)	24 (−5–28)		22 (10–36)	0.877
Correction angle (°)	9 (8–13)		9 (7–13)	0.233
mMPTA (°)	84 (83–86.6)		86 (82.5–88.2)	0.014
mLDFA (°)	89.5 (87–91)		87.6 (86–90)	0.001
JLCA (°)	4.0 (1.0–8.0)		4.0 (0–5.0)	0.368
KJLO (°)	−1.25 (−2.65 to 2.14)		−0.28 (−3.95 to 0.51)	0.993
Varus stress (°)	6.0 (4.0–7.0)		5.0 (2.0–9.0)	0.875
Valgus stress (°)	2.0 (1.0–3.0)		1.0 (−2.0–3.0)	0.371
%CTF (%)	5.0 (2.0–6.0)		6.0 (1.0–10.0)	0.129
Postoperative				
MA (%)	54 (41–56)		64 (57–67)	<0.001
mMPTA (°)	92 (90–94)		93 (91–97)	0.072
JLCA (°)	2.0 (1.0–3.0)		1.0 (0–3.0)	0.168
KJLO (°)	1.1 (0–8.0)		1.5 (0–8.0)	0.709
%CTF (%)	4.0 (0–5.0)		3.0 (−3.0 to 5.0)	0.329
Varus stress (°)	6.0 (3.0–9.0)		1.0 (3.0–8.0)	0.275
Valgus stress (°)	2.0 (1.0–3.0)		1.0 (0–2.0)	0.171

Abbreviations: JLCA, joint line convergence angle; KJLO, knee joint line obliquity; MA, mechanical axis; mLDFA, mechanical lateral distal femoral angle; mMPTA, mechanical medial proximal tibial angle.

No major complications were observed during the perioperative or follow‐up period. Specifically, there were no cases of infection or nonunion. All osteotomy sites demonstrated radiographic union within the expected timeframe.

Multiple linear regression analysis identified both preoperative mechanical mMPTA and mLDFA as significant predictors of postoperative MA percentage. A lower mMPTA was associated with a more medial postoperative MA (coefficient = −1.192, *p* = 0.010), as was a higher mLDFA (coefficient = −1.546, *p* = 0.006). Standardised coefficients indicated moderate predictive strength (*β* = −0.342 for mMPTA and *β* = −0.363 for mLDFA), with 95% confidence intervals excluding zero, confirming the statistical significance of these associations (Table 3). Both variables had negative coefficients, indicating that lower mMPTA and higher mLDFA values are associated with a more medial postoperative MA.

**Table 3:** Multiple regression analysis for factors associated with postoperative mechanical axis.

Postmechanical analysis (%)	Coefficient	Standard error	*β*	*p* value	95% CI
mMPTA (°)	−1.192	0.445	−0.342	0.010	−0.360 to −0.250
mLDFA (°)	−1.546	0.542	−0.363	0.006	−0.381 to −0.266

Abbreviations: mMPTA, mechanical medial proximal tibial angle; mLDFA, mechanical lateral distal femoral angle.

**Figure 3:**
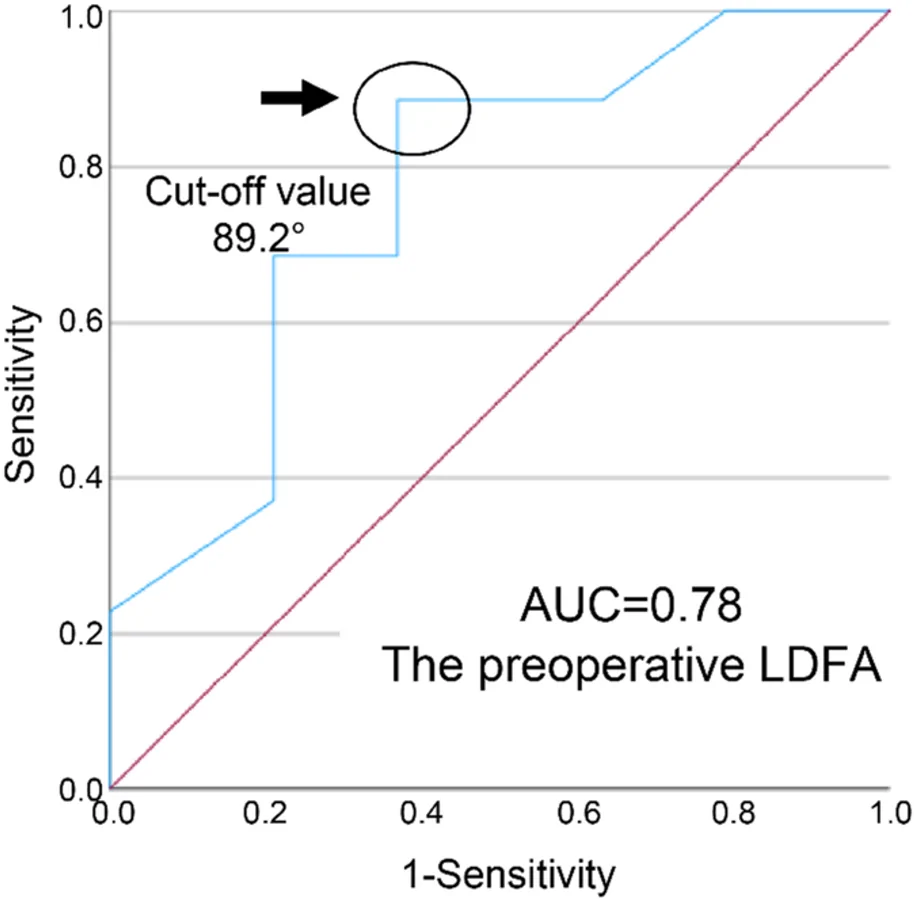
Receiver operating characteristic curve analysis. AUC, area under the curve; LDFA, lateral distal femoral angle.

The ROC curve analysis identified preoperative LDFA as a valuable predictor of undercorrection following MOWHTO. The optimal LDFA cut‐off value of 89.2° was determined from the point closest to the top‐left corner of the ROC curve, representing the best balance between sensitivity (36.8%) and specificity (88.6%), with an AUC of 0.78, indicating good discriminative ability (Figure 3).

**Figure 4:**
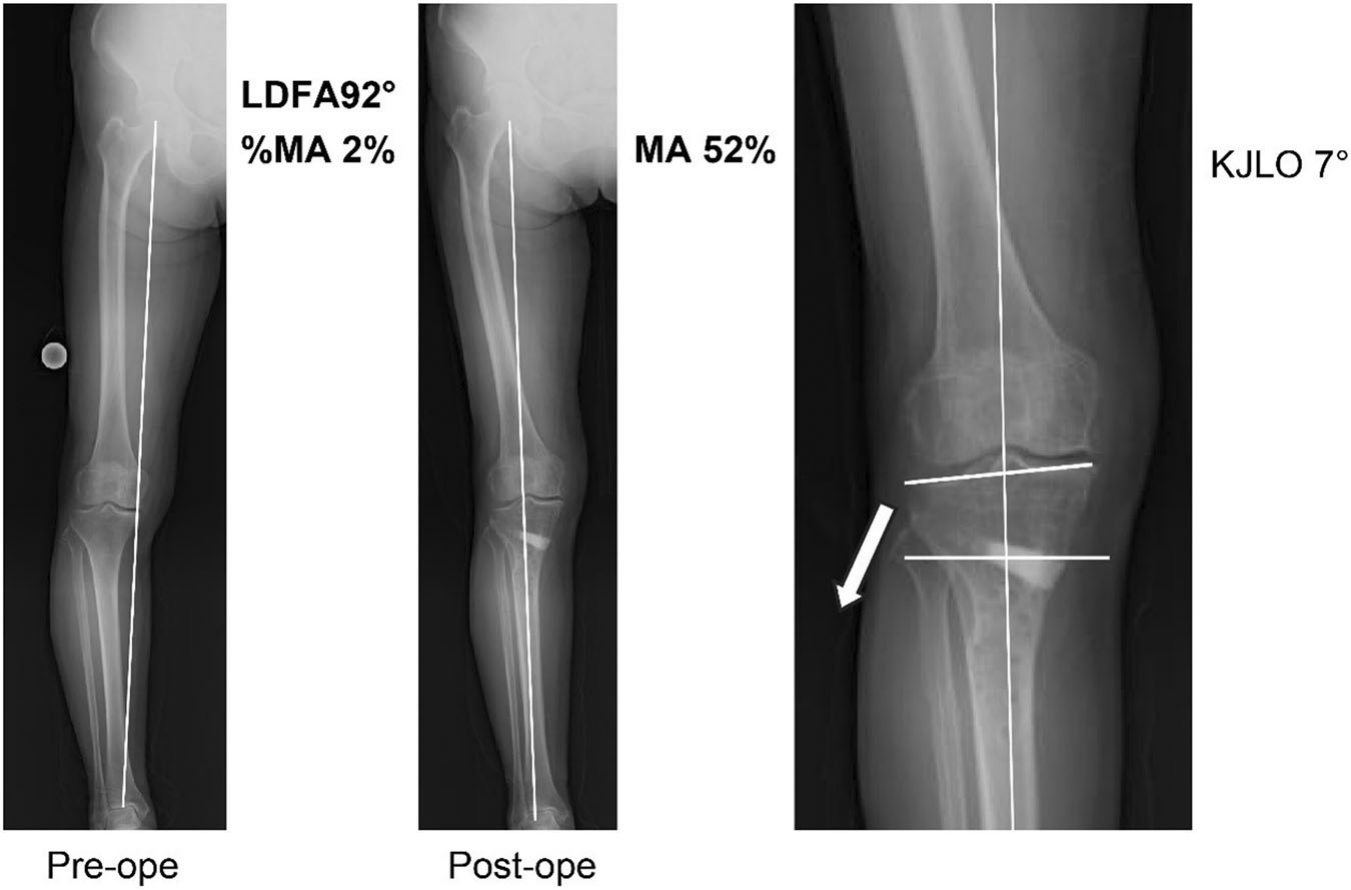
Radiographs demonstrating alignment correction pre‐ and post‐op from a representative case. Knee joint line obliquity (KJLO), lateral distal femoral angle (LDFA), mechanical axis (MA).

Based on the ROC curve analysis, patients were divided into two groups using a preoperative mLDFA threshold value of 89.2°. Fifteen patients had an LDFA ≥ 89.2°, and 39 had an LDFA < 89.2°. The group with LDFA ≥ 89.2° showed significantly higher BMI (*p* = 0.004), lower MPTA (*p* = 0.004) and greater JLCA (*p* = 0.007). Additionally, this group had significantly higher preoperative varus stress angle (*p* = 0.036), greater %CTF (*p* = 0.002) and lower pre‐ and postoperative KJLO (*p* = 0.022 and *p* = 0.030, respectively) (Table 4). Radiographic images of a representative case comparing preoperative and postoperative data are provided in Figure 4.

**Table 4:** Comparison of demographic and radiographic parameters stratified by preoperative mLDFA cut‐off value (89.2°).

	LDFA 89.2 or higher	LDFA less than 89.2	*p* value
*n* = 54	*n* = 15	*n* = 39	
Parameter			
BMI	27.6 (23.6–32.6)	25.7 (20.6–31.1)	0.004
MPTA (°)	84.0 (83.0–86.6)	86.0 (82.5–88.2)	0.004
JLCA (°)	4.6 (1.0–7.9)	3.4 (0.4–6.0)	0.007
Preoperative varus stress (°)	6.0 (4.0–9.0)	5.0 (2.0–7.0)	0.036
Preoperative % CTF (°)	6.0 (2.0–10)	4.0 (1.0–7.0)	0.002
Preoperative KJLO (°)	0.3 (−2.1 to 2.1)	0.97 (0–4.25)	0.022
Postoperative KJLO (°)	1.9 (0.5–8.1)	0.97 (0–4.25)	0.03

Abbreviations: BMI, body mass index; CTF, condylar tibial translation; JLCA, joint line convergence angle; KJLO, knee joint line obliquity; MPTA, medial proximal tibial angle.

## DISCUSSION

The most important finding of this study is that a high LDFA, specifically LDFA ≥ 89.2°, is significantly associated with undercorrection of lower‐limb alignment following MOWHTO. Our results suggest that in these cases, a postoperative phenomenon occurs resembling lateral displacement of the tibia, leading to increased KJLO and failure to achieve the intended valgus alignment. This undercorrection occurred despite achieving the targeted opening gap, suggesting that traditional correction planning may not fully account for complex deformity patterns involving distal femur.

Previous studies have reported similar challenges in patients with high LDFA, attributing undercorrection to either constitutional femoral varus morphology or degeneration of the medial femoral condyle in the context of advanced osteoarthritis [10, 11, 12]. However, the specific biomechanical mechanisms involved have remained ambiguous. Our study contributes to this by proposing that high LDFA leads to postoperative undercorrection. An increase in KJLO reflects this mismatch: while the ankle remains stationary, the proximal tibia tilts outward, resulting in an apparent lateral displacement. This leads to a discrepancy between the intended and achieved MA, ultimately contributing to undercorrection.

The role of KJLO has gained increasing attention in recent literature. Several studies have shown that excessive KJLO following OWHTO may persist for years and could influence long‐term clinical results [24]. While some authors argue that increased KJLO does not correlate directly with short‐term clinical outcomes, others have emphasised its importance as a marker of suboptimal alignment and joint line orientation [23]. Furthermore, excessive KJLO has been linked to preoperative lateral knee laxity [15], which may exacerbate varus thrust or instability, compounding the mechanical effects of femoral malalignment. Our findings are consistent with these observations, particularly in patients with high LDFA, where KJLO not only increases postoperatively but also contributed to mechanical axis deviation.

An important finding observed in our study was the significant difference in JLCA between patients with high versus low LDFA. While JLCA is often considered a marker of soft tissue imbalance or lateral laxity, our data suggest that a larger JLCA may also reflect underlying femoral deformity. In advanced osteoarthritis, wear of the medial femoral condyle can contribute to an apparent increase in LDFA, creating a scenario where bony and soft tissue factors simultaneously drive joint line asymmetry. This supports the view that in patients with high LDFA, deformity may be bifocal with contributions from both the femur and tibia.

These findings have direct clinical implications. In patients with bifocal varus deformity, isolated MOWHTO may be insufficient to restore optimal limb alignment. In such cases, DLO has been proposed as a more effective alternative, as it allows for correction at both the femur and tibia. Recent studies comparing DLO to OWHTO have demonstrated favourable clinical and radiographic outcomes, particularly in patients with severe deformity or abnormal femoral morphology [2]. Long‐term follow‐up studies have also confirmed that DLO provides sustained survival benefits without compromising future total knee arthroplasty outcomes [6]. While our study did not include femoral osteotomies, the consistent association between high LDFA and undercorrection suggests that DLO may be worth considering in selected cases to better distribute correction and reduce postoperative KJLO.

This undercorrection has been attributed to either constitutional femoral valgus morphology or degeneration of the medial femoral condyle with progression of osteoarthritis [10, 11]. However, the precise mechanism underlying this undercorrection has remained unclear. Our study provides novel insights by proposing a mechanical effect whereby high LDFA creates a situation in which the tibia appears to be ‘pushed laterally’, thereby disrupting intended alignment. An increase in KJLO suggests that the ankle joint remains relatively stationary while the proximal tibia tilts outwards. This creates an angular change indicating lateral displacement of the tibia, leading to a discrepancy between the planned and actual correction. Consequently, such lateral tilt can result in undercorrection of the MA, even when the planned osteotomy opening is technically accurate.

In patients with LDFA ≥ 89.2°, several preoperative parameters were significantly different compared to those with LDFA < 89.2°, including increased BMI, mechanical mMPTA, JLCA, preoperative varus stress angle, %CTF, and preoperative KJLO. Most of these parameters reflect more pronounced varus alignment, and the increase in postoperative KJLO observed in this group supports the hypothesis that improper joint line orientation contributes to undercorrection. The increased BMI in this group may suggest altered joint loading but warrants further investigation.

Given the above findings, isolated tibial correction may be insufficient in patients with a high LDFA. In such cases, it is essential to consider the possibility that the deformity lies primarily in the femur. Feucht et al. and Abe et al. have demonstrated that the location of the deformity varies across populations, with some showing femoral‐based deformity and others tibial‐based [1]. Akamatsu et al. further noted that DLO can better maintain joint line orientation compared to MOWHTO alone, especially in cases involving femoral deformity [3, 5]. Thus, our findings support considering femoral osteotomy, or DLO, in patients with LDFA ≥ 89.2° to achieve the desired postoperative alignment.

This study has a few limitations. First, although the sample size was relatively small, it was sufficient to achieve a statistical power of 0.8. Second, the lack of long‐term functional outcomes such as pain scores, return to activity, or patient satisfaction. Although this study focused on radiographic alignment, future studies should correlate these findings with clinical and functional outcomes. Third, only 2D imaging was utilised; 3D analysis could have offered more comprehensive spatial assessments. Fourth, the procedures were performed by multiple surgeons, and adjunct procedures, such as microfracture, were employed in cases involving meniscectomy or cartilage defects, potentially introducing variability in outcomes. Finally, the retrospective nature of the study and the relatively short follow‐up period of 6 months limit the ability to assess long‐term outcomes.

## CONCLUSIONS

High LDFA (≥89.2°) is a significant predictor of undercorrection following MOWHTO, likely due to inadequate valgization of the tibiofemoral joint and increased postoperative KJLO. These findings underscore the importance of preoperative planning that includes evaluation of femoral morphology. In cases where femoral varus deformity is present, alternative strategies such as DLO may be worth considering to optimise overall limb alignment. Future prospective studies and biomechanical modelling are warranted to elucidate further the mechanisms involved and validate surgical strategies for achieving optimal alignment.

## AUTHOR CONTRIBUTIONS

Akira Maeyama was responsible for data collection and drafting of the manuscript. Tetsuro Ishimatsu, Taiki Matsunaga, Kotaro Miyazaki, Yoshiaki Hideshima and Takuaki Yamamoto provided critical feedback, advice, and proofreading throughout the writing process. All authors reviewed and approved the final manuscript.

## CONFLICT OF INTEREST STATEMENT

The authors declare no conflicts of interest.

## ETHICS STATEMENT

This study was approved by the Institutional Review Board of Fukuoka University (approval no. H23‐10‐003). All participants provided informed consent prior to their inclusion in the study.

## Data Availability

Data sharing is not applicable to this article as no datasets were generated or analysed during the current study.
